# Fine-Mapping of *OvANS*: A Novel Gene Controlling White Flowers in *Orychophragmus violaceus*

**DOI:** 10.3390/biology14121669

**Published:** 2025-11-25

**Authors:** Yi Liu, Liwen Xie, Zichen Zhu, Chen Tan, Liwei Gao, Wenjie Shen, Shubei Wan, Xianhong Ge, Daozong Chen, Bin Zhu

**Affiliations:** 1 School of Life Sciences, Guizhou Normal University, Guiyang 550025, China; liuyi61@gnnu.edu.cn; 2 Ganzhou Key Laboratory of Greenhouse Vegetable, School of Life Sciences, Gannan Normal University, Ganzhou 341000, China; 19579879715@163.com (L.X.); cheng12342020@163.com (Z.Z.); tanchen2020@gnnu.edu.cn (C.T.); gaoliwei@gnnu.edu.cn (L.G.); shenwenjie@gnnu.edu.cn (W.S.); wanshubei@gnnu.edu.cn (S.W.); 3 National Key Laboratory of Crop Genetic Improvement, National Research Center of Rapeseed Engineering and Technology, Huazhong Agricultural University, Wuhan 430070, China; gexianhong@mail.hzau.edu.cn

**Keywords:** *Orychophragmus violaceus*, flower color, BSA-seq, RNA-seq, *OvANS*

## Abstract

*Orychophragmus violaceus* is an ornamental plant belonging to the Brassicaceae family, known for its profusion of white to light purple, pink, and purple flowers that bloom in early spring. However, there is a lack of reports regarding the localization of the genes responsible for flower color. The aim of this research was to locate the key genes controlling flower color variation in *O. violaceus*, providing loci for its genetic improvement of flower color. In this study, we constructed an F_2_ mapping population using the purebred white flower mutant line OvW-1 and the purebred purple flower line OvP-37, obtained candidate regions of the *Ovwf* locus using BSA-seq, and then used extreme recessive white-flowered plants for fine-mapping and combined with RNA-seq to predict key genes controlling the white-flowered trait. Genetic analysis indicated that the *Ovwf* trait is governed by a single recessive locus, designated as without cytoplasmic inheritance. Subsequently, BSA-seq and fine-mapping of the *Ovwf* locus within a 1.37 Mb interval. Gene annotation revealed that this interval encompasses 76 genes, of which only two genes—*OV03G032130* and *OV03G032280*—are potentially implicated in anthocyanin biosynthesis. Further RNA-seq analysis suggested that only *OvANS* (*OV03G032130*) was differentially expressed, which may be a critical gene influencing the white flower locus *Ovwf*. This is the first report of fine-mapping of the flower color locus in *O. violaceus*. These findings contribute valuable insights into the mechanisms underlying flower color variation in *O. violaceus* and present a promising target for the breeding of colorful varieties.

## Simple Summary

*O. violaceus* is one of the most prevalent flowering plants in early spring, characterized by petals that exhibit a range of colors, including white, light purple, and purple. However, there are currently no reports detailing the localization of genes responsible for flower color variation. Here, we constructed a mapping population by crossing a white-flowered mutant of *O. violaceus* with a purple-flowered wild-type. Utilizing bulked segregant analysis sequencing (BSA-seq) and map-based cloning techniques, we identified the key locus *Ovwf*, which regulates the white-flower trait. Integrating genome annotation data with RNA sequencing (RNA-seq) analysis, we determined that *OvANS* is the principal gene governing the white-flower trait. Our findings provide novel insights into the transcriptional regulation mechanisms underlying flower color in *O. violaceus* and present new targets for its genetic enhancement.

## 1. Introduction

*Orychophragmus violaceus*, the Chinese violet cress, belongs to the *Orychophragmus* genus within the Brassicaceae family. This species is native to China and is widely distributed across various ecological regions, and is abundant and also grows wild in Korea [1]. *O. violaceus* is utilized for ornamental purposes, as a petroleum source, as an ecological resource, for collection, and as food. The Chinese name of *O. violaceus*, Zhuge Cai, derives from the historical figure Zhuge Liang, who consumed this plant as a wild vegetable during his Northern Expedition in the Three Kingdoms period [1]. Due to its striking purple flowers, which bloom around early February, *O. violaceus* is also referred to as the February orchid (eryuelan). Its low-temperature tolerance and minimal water requirements make it suitable for cultivation as a winter cover crop in northern China [1]. The seeds of *O. violaceus* are rich in oil and contain a high level of unsaturated fatty acids, and they are cultivated as a potential industrial oilseed crop, particularly because of their abundance of 24-carbon dihydroxy fatty acids (diOH-FAs), which contribute to their excellent lubrication properties at high temperatures [2]. Furthermore, recent studies have demonstrated that *O. violaceus* contains metabolites such as flavonoids, alkaloids, phenylpropanoids, phenolic acids, and terpenes, which exhibit various pharmacological activities, including antioxidant, anti-radiation, anti-tumor, hepatoprotective, anti-ferroptosis, anti-inflammatory, and antibacterial properties [3]

In nature, plant pigments are primarily classified into chlorophylls, carotenoids, anthocyanins, and betaines, which contribute to the richness and diversity of plant colors. Anthocyanins, as water-soluble flavonoid secondary metabolites, produce color variations ranging from yellow to orange, red, purple, and blue, and possess antioxidant properties [4]. Previous studies have established that the synthesis and accumulation of anthocyanins in the petals of *O. violaceus* are crucial for its purple coloration [5,6,7]. The biosynthesis pathway and transcriptional regulation mechanisms of anthocyanins have been well characterized in species such as *Arabidopsis thaliana* [8] *chrysanthemum* [9], and grape (*Vitis vinifera* L.) [10]. The anthocyanin biosynthesis pathway primarily involves the synthesis of phenylpropanoid compounds, flavonoids, and the subsequent modification of anthocyanins. The expression of genes encoding proteases that catalyze the synthesis of these metabolites is primarily regulated by MYB transcription factors or their MBW complexes, which include MYB and basic helix–loop–helix (bHLH) transcription factors and TRANSPARENT TESTA GLABRA1 (TTG1), a WD repeat protein [11,12]. Numerous studies have focused on the transcriptional regulation of anthocyanins in closely related species of *O. violaceus*, including closely related genus species *Brassica rapa*, *B. oleracea*, *B. napus*, and *B. juncea*, identifying key regulatory genes such as *BoMYB2*, *BrMYB2*, *BnaPAP2.A7*, and *BjPur* [13,14,15,16].

The release of the chromosome-level genome of *O. violaceus* [17,18] has provided new opportunities for exploring the mechanisms underlying flower color variation. Utilizing the high-quality genome map of *O. violaceus*, we previously conducted a systematic identification of genes involved in anthocyanin biosynthesis genes (ABGs). All ABGs have been thoroughly characterized within the *O. violaceus* genome [6]. The anthocyanin biosynthesis mechanism in *O. violaceus* likely adheres to a transcriptional regulatory mechanism akin to that observed in other plants, where the expression of structural genes is primarily regulated by transcription factors [6,7]. However, there have been no published reports on the mapping of genes regulating flower color in *O. violaceus*. Flower color is one of the most important agronomic traits of *O. violaceus*. By studying the inheritance patterns of genes that control white flowers, breeders can develop new varieties of *O. violaceus* in a targeted manner, thereby improving its ornamental value and economic benefits.

To fill the gap, we constructed an F_2_ mapping population and used BSA-seq, fine-mapping, and RNA-seq to determine the potential candidate gene for white flower *O. violaceus*, providing loci for its genetic improvement of flower color. The genetic analysis indicated that the white flower locus is controlled by a single recessive locus, without cytoplasmic inheritance. Bulked-segregant analysis sequencing (BSA-seq) analysis revealed that the locus responsible for the white flower locus, designated *Ovwf*, is located within the 9.69–15.00 Mb interval on chromosome Ov03. Subsequently, by utilizing white-flowered individuals from an F_2_ and BC_1_F_1_ segregating population and employing map-based cloning techniques, we localized the *Ovwf* locus to a 1.37 Mb interval on chromosome Ov03, which encompasses 76 genes. Furthermore, RNA-seq analysis of multiple *O. violaceus* flower petals showed that only *OV03G032130* (*OvANS*) among 76 genes was differentially expressed in white, light purple, and purple flowers. Therefore, we hypothesize that *OvANS* is a key gene regulating the white flower trait in *O. violaceus*. This is the first gene to be mapped in *O. violaceus*, providing an excellent target for investigating the mechanisms regulating flower color and for genetic breeding in this species.

## 2. Materials and Methods

### 2.1. Plant Materials

Two purebred lines of wild-type *O. violaceus* (purple flower, OvP-37) and white flower mutant (OvW-1) were used in reciprocal crossing. The reciprocal hybrid F_1_ plants were self-pollinated to produce an F_2_ segregating population. Simultaneously, the F_1_ plants were backcrossed with OvW-1 to produce a BC_1_F_1_ segregating population. All plants were grown from seeds and cultivated in an experimental base at Gannan Normal University, Ganzhou, China, adopting the field planting and management model of Brassicaceae crops. The seeds can be obtained from the authors on request.

### 2.2. Statistical Analysis

A goodness-of-fit test for segregation analyses of the anthracnose reaction was performed with the chi-square (χ^2^) test using the Genes software online (https://arquivo.ufv.br/genetica/WebSite1/Default.aspx, accessed on 19 November 2025) [19]. In the F_2_ segregating population, the Mendelian segregation hypothesis was a 3:1 (3 purple flower–1 white flower) ratio. In the BC_1_F_1_ segregating population, the Mendelian segregation hypothesis was a 1:1 (1 purple flower–1 white flower) ratio.

### 2.3. BSA-Seq Analysis

For fine-mapping the *white flower* (*Ovwf*) locus of *O. violaceus*, the parents (OvP-37 and OvW-1), 30 extreme purple and white flower buds in the F_2_ segregating population plants at the flowering stage were collected, immediately frozen in liquid nitrogen, and stored at −80 °C for BSA-seq. The genomic DNA was extracted using a modified CTAB method [20]. The extracted genomic DNA was evaluated for quality and quantity using the NanoDrop One spectrophotometer (Thermo Fisher, Waltham, MA, USA) and the Qubit 3.0 fluorometer (Thermo Fisher, Waltham, MA, USA). Then, the extreme purple and green DNA mix pools were obtained by adding equal quantities of DNA from 30 extreme purple flower F_2_ plants (purple-pool) and 30 white flower F_2_ individuals (white-pool), along with two parents (P1- and P2-pool) for BSA-seq. The extracted genomic DNA was evaluated for quality and quantity using the NanoDrop One spectrophotometer and the Qubit 3.0 fluorometer. The qualified DNA was then used to construct NGS libraries and subjected to 350 bp paired-end sequencing on the DNBSEQ-T7 platform (Huada, Shenzhen, China).

The raw data of four pools were purified using fastp software v 0.23.0 [21] to remove adapters and low-quality bases to get clean data, and the length of reads must be greater than 50 bp. The clean data were aligned to the *O. violaceus* reference genome [18] using the Burrows–Wheeler Alignment tool (BWA) v0.7.15-r1140 [22] to generate SAM files, which were sorted and converted to BAM format using SAMtools v1.3.1 [23]. Then, GATK v3.7 [24] was used to perform tasks including duplicate read removal, local realignment, and base quality score recalibration. We used QTLseqr v.0.7.5.2 [25] to calculate the Δ(SNP-index) value for each variant site and examined its distribution across the genome using a 2 Mb sliding window. Additionally, 95% confidence intervals (CIs) were calculated to identify the target region controlling the white flower in *O. violaceus*.

### 2.4. Fine-Mapping of Ovwf Locus

Then, the flower buds of 295 white flower plants from the F_2_ segregating population and 247 white flower plants from the BC_1_F_1_ segregating population were collected, immediately frozen in ice, and stored at −20 °C for DNA extraction. Based on the insert and delete information in the candidate region, we designed 30 pairs of specific InDel (insert or delete) primers (Supplementary Table S1) in two separate steps and used the map-based cloning method to locate the *Ovwf* locus.

### 2.5. RNA Sequencing Data Analysis

To predict candidate genes for the *Ovwf* locus, we collected *O. violaceus* white (OvW), light purple (OvLP), and purple (OvP) flower petals RNA-seq data (PRJNA1136702) from NCBI (https://www.ncbi.nlm.nih.gov/), and we previously published *O. violaceus* OvP-37 and OvW-1 flower petals RNA-seq data (PRJNA1094639). We used Trimmomatic software v0.39 [26] to trim the paired-end reads, removing adaptors and low-quality reads. Clean reads were then aligned to the *O. violaceus* reference genome [18] using HISAT2 software v2.1.0 [27] with default settings. We calculated read counts and gene expression levels in FPKM (Fragments Per Kilobase Million) for each gene using StringTie based on gene length. TBtools-II software v0.665 [28] was used to create a heatmap of gene expression levels related to the anthocyanin biosynthesis pathways with Z-score. For significantly differentially expressed gene (DEG) analysis in each group, we used DESeq2 v4.5 [29], genes with *p*-value ≤ 0.01 and Log_2_FC ≥ 2 were assigned as DEGs. These DEGs were further subjected to enrichment analyses using the KEGG (https://www.genome.jp/kegg/) and GO (https://geneontology.org/) websites.

## 3. Results

### 3.1. The Phenotype of O. violaceus

**Figure 1:**
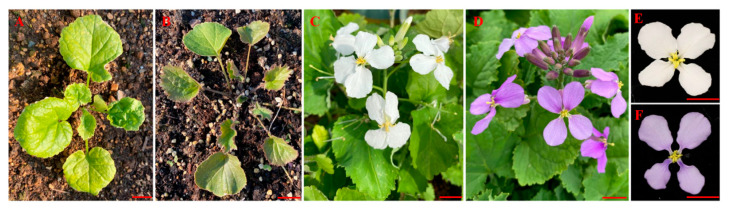
Phenotypic characteristics of the white flower mutant and wild-type *O. violaceus*: (**A**) *O. violaceus* white flower mutant at the seedling stage. (**B**) *O. violaceus* purple flower (wild-type) at the seedling stage. (**C**) *O. violaceus* white flower mutant at the flowering stage. (**D**) *O. violaceus* purple flower at the flowering stage. (**E**) Single flower phenotype of the white flower *O. violaceus* mutant. (**F**) Single flower phenotype of the purple flower *O. violaceus*. Scale bar, 2 cm.

The white flower (wf) mutant of *O. violaceus*, a natural variant derived from a purple flower population, exhibits notable differences throughout its growth stages. During the seedling phase, the leaves of the wf mutant are less notched compared to those of the wild-type and display a pale yellow coloration under low temperatures (Figure 1A). In contrast, the wild-type *O. violaceus* exhibits a purple hue attributed to the accumulation of anthocyanins in its leaves and petioles (Figure 1B). During the flowering stage, the petals of the wf mutant are pure white (Figure 1C), whereas the petals of the wild-type acquire a purple hue due to the synthesis and accumulation of anthocyanins (Figure 1D). Further analysis of individual flower phenotypes revealed no significant differences between the wf mutant and the wild-type, apart from the variation in petal color (Figure 1E,F).

### 3.2. A Single Recessive Locus wf Controls the White Flower Phenotype in O. violaceus

To analyze the inheritance mechanism of the wf mutant, we conducted reciprocal crosses between a purebred line of wild-type purple-flowering *O. violaceus* (OvP-37) and the wf mutant (OvW-1) to generate reciprocal hybrids. All F_1_ plants exhibited purple flowers, indicating that the wf locus is not influenced by cytoplasmic genetic effects. Subsequently, the F_1_ plants were self-pollinated and backcrossed with the OvW-1 parent to construct F_2_ and BC_1_F_1_ segregating populations. Among the 1224 F_2_ segregating individuals, 929 exhibited purple flowers and 295 displayed white flowers, yielding a segregation ratio of 3:1 (χ^2^ = 0.48) (Table 1). In the 508 BC_1_F_1_ segregating individuals, 261 exhibited purple flowers and 247 displayed white flowers, resulting in a segregation ratio of 1:1 (χ^2^ = 0.33) (Table 1). These results suggest that the *wf* locus is controlled by a recessive locus.

### 3.3. The wf Locus Is Located on Chromosome Ov03 by BSA-Seq Analysis

**Figure 2:**
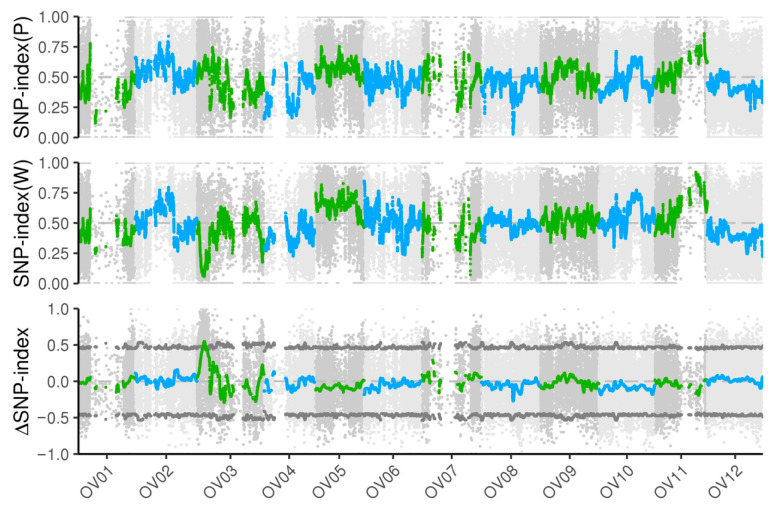
SNP-index and ΔSNP-index plot from BSA-seq. The grey dashed line indicates the threshold generated by the 95% confidence interval.

To identify the key locus associated with the wf mutant, we constructed four pools comprising extreme purple flowers, white-flowered plants, and both parental lines from the F_2_ segregating population for BSA-seq analysis. The results indicated that the SNP index of the white-pool (W) exhibited a significantly downregulated SNP peak on chromosome Ov03 and a significantly upregulated SNP peak on chromosome Ov11 (Figure 2). Similarly, the SNP index of the purple-pool (P) also displayed a significantly upregulated SNP peak on chromosome Ov11 (Figure 2). Furthermore, the ΔSNP index for both the white and purple pools revealed a significantly upregulated SNP peak exclusively on chromosome Ov03 (Figure 2), thereby reinforcing the conclusion that the wf mutant is regulated by a single locus located on chromosome Ov03.

### 3.4. The Ovwf Locus Was Fine-Mapped to a 1.37 Mb Region

**Figure 3:**
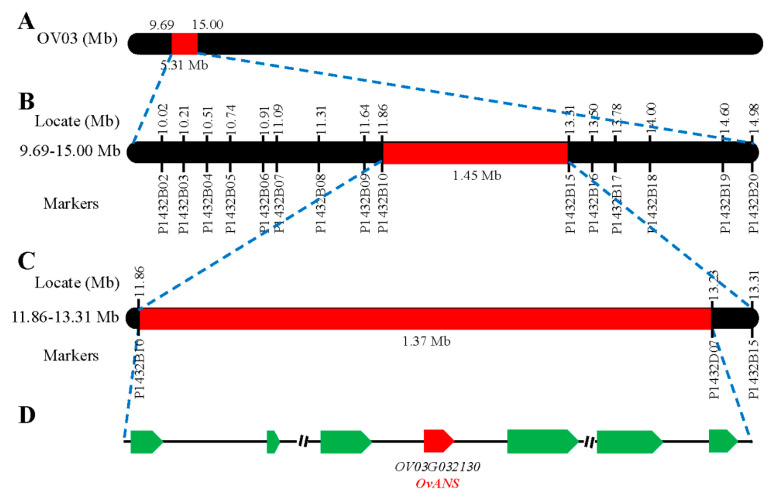
Fine-mapping the white flower traits of *O. violaceus*: (**A**) BSA-seq analysis of candidate intervals of the locus controlling white flowers. (**B**) Initial mapping of the white flower locus to a 1.45 Mb region between the markers P1432B10 and P1432B15. (**C**) Fine-mapping of the white flower locus to a 1.37 Mb region. (**D**) The candidate genes in the fine-mapping region.

To fine-map the *Ovwf* locus, we designed 30 pairs of indel markers (Supplementary Table S1) using Primer 3, based on sequence insertions and deletions within the 9.69–15.00 Mb interval on chromosome Ov03 (Figure 3A) obtained through BSA-seq analysis. Initially, we utilized white-flowered plants from F_2_ and BC_1_F_1_ segregating populations to narrow the candidate interval to a 1.45 Mb region between markers P1432B10 and P1432B15 (Figure 3B). Subsequently, we developed additional markers to further reduce the target interval to a 1.37 Mb region between markers P1432B10 and P1432D07 (Figure 3C). The remaining interval could not be further narrowed due to the lack of recombinant exchange plants.

### 3.5. OvANS Was Predicted to Be the Candidate Gene for the Ovwf Locus

We further analyzed the gene distribution within the target interval using reference genome annotation information, revealing a total of 76 genes within the 1.37 Mb interval (Supplementary Table S2). Subsequently, we conducted a joint analysis utilizing previously published petal RNA-seq data from OvW-1 and OvP-37, alongside white (OvW), light purple (OvLP), and purple (OvP) varieties obtained from NCBI. We analyzed the expression patterns of 76 genes within the target region, and 52 genes showed different abundances of expression (Supplementary Table S3). The differential expression analysis results showed that only *OV03G031980* and *OV03G032130* exhibited differential expression between the OvW-1 vs. OvP-37 and OvW vs. OvP groups. Notably, only *OV03G032130* demonstrated differential expression across all three groups: OvW-1 vs. OvP-37, OvW vs. OvLP, and OvW vs. OvP (Supplementary Tables S4–S6). Annotation results revealed that *OV03G032130* encodes leucoanthocyanidin dioxygenase (ANS/LDOX), which we designated as *OvANS*. We hypothesized that *OvANS* plays a crucial role as a key gene controlling the *Ovwf* locus. Our previous quantitative reverse transcription polymerase chain reaction (qRT-PCR) analysis demonstrated that the *OvANS* gene is differentially expressed in the petals of OvW-1 and OvP-37, suggesting its potential role as a key regulator of the white flower trait in *O. violaceus*. Cloning of the full-length *OvANS* gene sequence, encompassing both the promoter and gene regions, in OvW-1 and OvP-37 revealed that, aside from two significant insertions in the promoter region of OvW-1, the sequences of the other regions were identical [6]. Building on our previous reports and the findings of this study, we further affirm that *OvANS* is a crucial gene governing the white flower trait in *O. violaceus*, as variations in the *OvANS* promoter region result in its inactivation.

**Figure 4:**
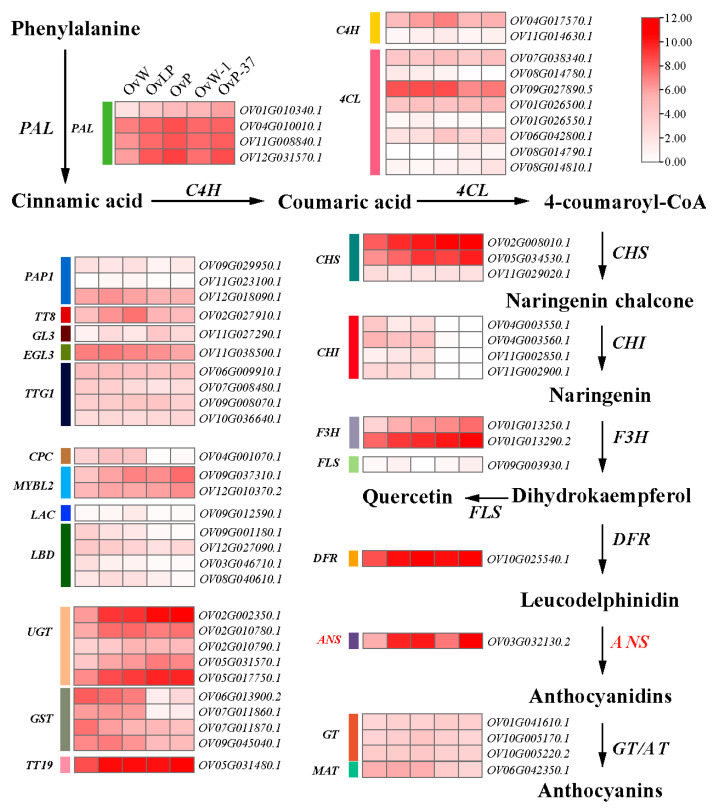
ABGs expression patterns of different colors of flower petals in *O. violaceus*. Detailed information and data can be found in Supplementary Table S5.

To further analyze the expression pattern of *OvANS* in the anthocyanin biosynthesis pathway and to explore its crucial role in flower color regulation, we generated a heatmap of ABG expression patterns using data from five petal groups (Figure 4; Supplementary Table S7). The results indicated that the genes involved in the phenylpropanoid biosynthesis pathway, specifically *PAL*, *C4H*, and *4CL*, were expressed in both white and purple petals; however, their expression levels were significantly elevated in purple petals compared to white ones. The expression patterns of the flavonoid biosynthesis pathway genes, including *CHS*, *CHI*, and *F3H*, exhibited some variability, with *CHI* expression being notably low overall. The *FLS*, which catalyzes flavonol biosynthesis, was nearly undetectable in all petal types. In contrast, the genes associated with anthocyanin biosynthesis and modification, *DFR*, *ANS*, *UGT*, *GST*, *SGT*, and *MATE*, were expressed at significantly higher levels in purple flowers than in white flowers, with *OvANS* showing particularly high expression in light purple and purple petals. Furthermore, the positive regulatory transcription factors *PAP1*, *TT8*, *GL3*, *EGL3*, and *TTG1* were generally upregulated in purple petals. The expression patterns of the negative regulatory transcription factors *CPC*, *MYBL2*, *LAC15*, and *LBD37/38/39* mirrored those of the positive regulatory factors, potentially due to negative feedback regulation stemming from the synthesis and accumulation of anthocyanins.

## 4. Discussion

*O. violaceus* is a native species of China, characterized by its long flowering period, elegant colors, and resilience to cold, drought, and barren conditions. As one of the most prevalent flowers in early spring, *O. violaceus* is extensively cultivated in Chinese gardens and parks, resulting in vibrant floral displays. Notably, the unassuming *O. violaceus* frequently appears in literary works as a symbol of grassroots resilience, thereby acquiring a unique cultural identity. However, due to its large genome size and complex genetic composition, studies on the flower color of *O. violaceus* have been scarce. Following the successful sequencing of the *O. violaceus* genome, we utilized this genomic information to explore the molecular mechanisms that govern its flower color.

In our previous study, we conducted a comparative RNA-seq analysis using the white-flowered mutant OvW-1 and the purple-flowered wild-type OvP-37 of *O. violaceus*. Based on the identification and expression pattern analysis of ABGs, we hypothesized that *OvANS* may be a key gene regulating flower color. We successfully cloned the full-length *OvANS* sequence from both OvW-1 and OvP-37, discovering that two insertions in the promoter region of the white flower variant may be responsible for the silencing of *OvANS* [6]. Notably, a combined transcriptome and metabolome analysis of petals from white, light purple, and purple varieties of *O. violaceus* revealed that the expression levels of *OvPAL*, *OvCHS*, *OvCHI*, *OvF3′H*, *OvDFR*, *Ov3MaT1*, *OvMT*, *OvUFGT*, and *OvANS* increased with the intensity of flower color. This led the authors to speculate that *OvANS* plays a pivotal role in flower color regulation [7] However, while reverse genetics methods can confirm that overexpression of the ABG pathway contributes to darker flower color, they do not identify the specific genes responsible for color variation. Consequently, in this study, we constructed F_2_ and BC_1_F_1_ genetic populations using purebred lines OvW-1 and OvP-37. Genetic analyses indicated that the white flower trait is governed by a single recessive locus, designated *Ovwf*. We subsequently employed BSA-seq and map-based cloning techniques to locate the key gene, *OvANS*, at the *Ovwf* locus, with its functional loss accounting for the white flower phenotype. Further comparative analysis of RNA-seq data from multiple *O. violaceus* petals and investigation of ABGs expression patterns corroborated that *OvANS* is indeed the key gene controlling the white flower trait. Based on our previous report [6] and the findings of the current study, we further confirm that *OvANS* is a critical gene regulating the white flower trait of *O. violaceus*, as variations in the promoter region of *OvANS* result in its inactivation. This research provides new insights into the mechanisms underlying flower color development in *O. violaceus* and identifies potential targets for breeding vibrant and colorful flower varieties.

In the *Brassica* genus, a close relative of *O. violaceus*, numerous genes regulating leaf, petal, and seed coat color have been identified. Notable genes influencing leaf color in *B. rapa* include *BrMYB2* [15], *BraANS.A03* [30], and *BraCHI* [31]. In *B. oleracea*, *BoMYB2* [13] plays a significant role in leaf coloration, while *BnaA.PL1* [32], *BnaPAP2.A07* [14,33], and *BjMYB113* [34] are key genes for leaf color regulation in *B. napus*. Research on flower color variation has predominantly concentrated on *B. napus*, with *BnaC3.CCD4* [35] identified as a crucial gene for the regulation of white and yellow flowers. Additionally, *BnaA07.PAP2* [36], *BnaPAP2.A07* [33], *MYB75* [37], *miR156-SPL9*, and *miR828-PAP2* regulatory modules [38] and *RUBY* [39] are essential for the formation of orange, orange-red, and purple flowers, respectively. The enzyme ANS plays a crucial role in the catalysis of anthocyanin synthesis, with its homologous being pivotal in regulating anthocyanin biosynthesis in these *Brassica* crops [13,14,15,30,33,36]. This suggests the presence of highly complex anthocyanin transcriptional regulatory mechanisms in various *Brassica* crops, whereas similar mechanisms in the leaves, petals, and mesocarp of *O. violaceus* remain unreported.

Notably, *O. violaceus* serves as a significant source of genes for enhancing flower color in closely related *Brassica* crops. Hu et al. (2002) [40] reported that intergeneric hybrids between *B. napus* and *O. violaceus* exhibited partially orange-red petals. Subsequently, researchers identified new germplasm with orange-red flowers derived from intergeneric somatic hybrids between *B. napus* and *O. violaceus* [41,42]. Furthermore, we successfully transformed *OvPAP2*, a gene cloned from *O. violaceus*, into *B. napus*, resulting in the generation of a novel *B. napus* germplasm characterized by red flowers. This represents the first report of a colored-flowering rapeseed germplasm developed through genetic engineering in rapeseed [43]. These studies further enhance the application value of *O. violaceus* and open new avenues for identifying key genes that regulate anthocyanin biosynthesis, understanding the transcriptional regulatory mechanisms of anthocyanins in plants, and developing new anthocyanin-rich germplasm resources.

## 5. Conclusions

*O. violaceus* is an exceptionally cold-tolerant early spring jungle flower, characterized by its vibrant purple blooms that create a highly ornamental spectacle. In this study, we constructed a mapping population by crossing a white-flowered mutant with a purple-flowered wild-type. By integrating BSA-seq, map-based cloning, and functional annotation, we identified *OvANS* as a potential candidate gene responsible for the white-flower trait. Subsequent analyses of multiple RNA-seq datasets further confirmed that *OvANS* is a key gene regulating the white-flower trait in *O. violaceus*. This discovery enhances our understanding of the function of *OvANS*, elucidates the regulatory node genes involved in flower color formation in *O. violaceus*, lays a solid foundation for further research into the molecular mechanisms of anthocyanin biosynthesis, and provides a theoretical framework for the genetic improvement of flower color in *O. violaceus*.

## Figures and Tables

**Table 1:** Genetic analysis of *wf* loci in *O. violaceus*.

Type	Population	Purple	White	Theoretical Ratio	Actual Ratio	χ^2^
F_2_	1224	929	295	03:01	3.15:1	0.48
BC_1_F_1_	508	261	247	01:01	1.06:1	0.33
Note: χ^2^ (0.05, 1) = 3.84.						

## Data Availability

Data were derived from public domain resources. The data presented in this study are available in NCBI at https://www.ncbi.nlm.nih.gov/, reference numbers PRJNA1094639 and PRJNA1136702.

## Supplementary Materials

The following supporting information can be downloaded at: https://www.mdpi.com/article/10.3390/biology14121669/s1, Table S1. Primer informations for fine mapping. Table S2. Functional annotation of candidate interval genes. Table S3. Candidate interval genes expression patterns. Table S4. Differentially expressed genes and their expression levels in OvP-37 and OvW-1 petals of *O. violaceus*. Table S5. Differentially expressed genes and their expression levels in OvLP and OvW petals of *O. violaceus*. Table S6. Differentially expressed genes and their expression levels in OvP and OvW petals of *O. violaceus*. Table S7. Expression patterns of genes related to anthocyanin biosynthesis.

## Abbreviations

PAL: phenylalanine ammonia-lyase; C4H: cinnamate-4-hydroxylase; 4CL: 4-Coumarate CoA ligase 4; CHS: chalcone synthase; CHI: chalcone isomerase; F3H: flavanone 3-hydroxylase; FLS: flavonol synthase; DFR: dihydroflavonol 4-reductase; ANS: anthocyanidin synthase; UFGT: UDP-flavonoid glucosyltransferase; GST: glucosyltransferase; MATE: multidrug and toxic extrusion compound transporters; TT8/19: transparent testa8/19; PAP1: production of anthocyanin pigment 1; GL3: glabra 3; EGL3: enhancer of glabra 3; TTG1: transparent testa glabra 1; CPC: caprice; MYBL2: Arabidopsis myb-like 2; LAC15: laccase-like 15; LBD 37/38/39: asymmetric leaves2-like 37/38/39.
